# First record of the marbled ray, *Dasyatis marmorata* (Elasmobranchii: Dasyatidae), from Greece (central Aegean Sea)

**DOI:** 10.3897/BDJ.8.e51100

**Published:** 2020-04-13

**Authors:** Archontia Chatzispyrou, Chrysoula Gubili, Maria Laiaki, Danai Mantopoulou-Palouka, Stefanos Kavadas

**Affiliations:** 1 Hellenice Centre for Marine Research, Institute of Marine Biological Resources and Inland Waters, Argyroupoli, Athens, Greece Hellenice Centre for Marine Research, Institute of Marine Biological Resources and Inland Waters, Argyroupoli Athens Greece; 2 University of Patras, Department of Biology, Rio Patras, Greece University of Patras, Department of Biology Rio Patras Greece; 3 Hellenic Agricultural Organization-DEMETER-Fisheries Research Institute, Nea Peramos, Kavala, Greece Hellenic Agricultural Organization-DEMETER-Fisheries Research Institute, Nea Peramos Kavala Greece

**Keywords:** biometrics, Dasyatidae, DNA barcoding, Eastern Mediterranean Sea, geographic range.

## Abstract

**Background:**

Currently, seven dasyatid species have been described in the Mediterranean Sea: *Bathytoshia lata*, *Dasyatis marmorata*, *Dasyatis pastinaca*, *Dasyatis tortonesei*, *Himantura uarnak*, *Pteroplatytrygon violacea* and *Taeniura grabata*. Papaconstantinou (2014) listed four species of Dasyatidae occurring in Greece (*P. violacea*, *D. pastinaca*, *D. tortonesei* and *D. centroura*; the latter was a case of misidentification and it is currently identified as *B. lata*, according to genetic analysis). However, the marbled stingray (*D. marmorata*) was not amongst them. Here, the presence of *D. marmorata* was examined for the first time in Greece.

**New information:**

The present study provides updated information on the geographical distribution of *D. marmorata* in the Eastern Mediterranean Sea. A juvenile male stingray was captured in February 2019, during an onshore survey in Maliakos Gulf, located in the central Aegean Sea, Greece. The ray was examined at the Fisheries laboratory of the Hellenic Centre for Marine Research (HCMR) in Athens and was identified as *D. marmorata*. Morphological characters were recorded and DNA barcoding was applied to confirm the species identification. The combination of the two methods verified the occurrence of the marbled ray in the Greek waters. This is the first record of *D. marmorata* from the Aegean Sea.

## Introduction

Although batoids are a key group of chondrichthyan fish, nevertheless considerable taxonomic uncertainties exist for many taxa, due to the lack of useful diagnostic morphological characters (Last et al. 2016a). Unfortunately, molecular identification studies are scarce and many field guides do not accurately reflect the rapidly changing pace of batoid taxonomy. Currently, seven species belonging to the family Dasyatidae have been described in the Mediterranean Sea, which is the result of considerable updating of species occurrence in the region; for example, the roughtail stingray, valid as *Dasyatis centroura* Mitchill, 1815, that was previously reported inhabiting the Mediterranean Sea, is now considered to be solely distributed along the Western coasts of the Atlantic Ocean, whereas its closely related species, the brown stingray *Bathytoshia lata* Garman, 1880, occupies the Mediterranean and the eastern coasts of Africa (Last et al. 2016b).

The marbled stingray, *Dasyatis marmorata* Steindachner, 1892, is a newly-recorded species in the north-eastern Mediterranean (Yeldan and Gundogdu 2018), despite its presence in the southern part of the sea since 1993 (Cowley and Compagno 1993). Previous records were confined to Tunisia (Capapé and Zaouali 1993, Capapé and Zaouali 1995), Israel (Golani and Capapé 2004) and, more recently, in Turkey. Its presence in the Turkish waters has been confirmed in Adana, Mersin, Iskenderun Bay (Bilecenoglu in Kapiris et al. 2014, Erguden et al. 2014, Yeldan and Gundogdu 2018) and in the Gulf of Antalya (Özgür Özbek et al. 2015). The first three areas report the species distribution at depths < 50 m, indicating its preference to shallower waters; nevertheless, it was also captured between 50 and 100 m in the Gulf of Antalya. This suggests that it occupies deeper waters, whilst shallow waters may be used as nursery grounds (Özgür Özbek et al. 2015).

Sexual maturity in *D. marmorata* is generally achieved at 30 cm and 32 cm in Disc Width (DW) for males and females, respectively and the gestation period lasts around three to four months, with litter size of two to four embryos (Bradai et al. 2012). It is occasionally caught as by-catch in the Mediterranean Sea. Scattered observations of the species along the Eastern basin may indicate an occasional occurrence; however, this could suggest that the misidentification of the marbled ray is common (Bilecenoglu in Kapiris et al. 2014, Erguden et al. 2014). The species is also listed as ‘Data Deficient’, both in the Mediterranean Regional Red List (Notarbartolo di Sciara et al. 2009) and the Global Red List of IUCN (Bradai et al. 2016).

*Dasyatis marmorata* is closely related to the sympatric species *Dasyatis pastinaca* Linnaeus, 1758, which could have caused errors and/or confusion in the identification of these species in the past. They share common external features and can be mainly distinguished by the disc length to disc width ratio (Cowley and Compagno 1993) and the colouration on the dorsal surface of the pectoral fins (Yeldan and Gundogdu 2018). Moreover, *D. marmorata* exhibits a yellowish surface with blue blotches, whereas *D. pastinaca* has a uniformly dark brown to olive or grey colouration. Tail spines can also be used as a species identification tool since these congenerics differ in length and serrations of this structure (Schwartz 2007). Additionally, differences in the length of the dorsal and ventral tail fold have been verified and proposed by Erguden et al. 2014.

This is the first report of the marbled stingray in Greek waters, identified macroscopically and verified through molecular analysis. This observation will allow us to update its distribution in the Eastern Mediterranean.

## Materials and methods

**Figure 1.**
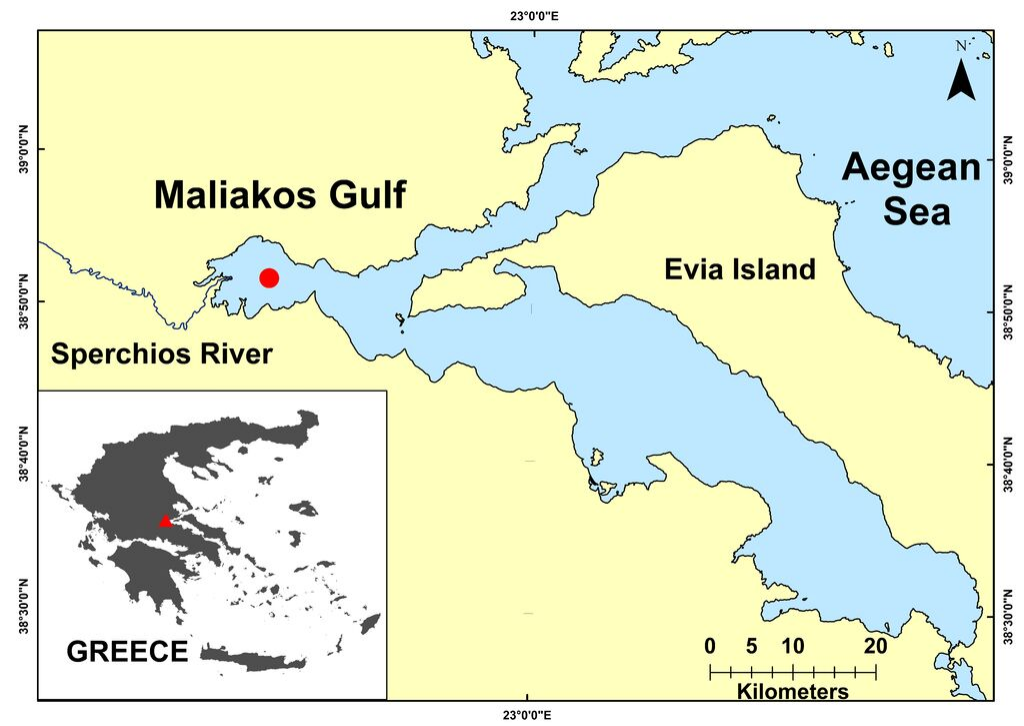
The collection site (Maliakos Gulf) of *D. marmorata* in the central Aegean Sea, Greece.

A male juvenile specimen of *D. marmorata*, measuring 188 mm in DW, was caught on 09/02/2019, in Maliakos Gulf (central Aegean Sea) (Fig. 1). The mean temperature of the gulf was recorded at 12.97˚C and the mean salinity value was 35.89 PSU. Maliakos Gulf is a semi-enclosed embayment, located on the central west mainland of Greece, receiving water form Spercheios River that flows into the inner part of the gulf and is characterised as a high productivity area (Kormas et al. 2002). The occurrence of batoids in this gulf has been under investigation through several HCMR scientific surveys (Kavadas and Siapatis in Ζenetos et al. 2015). The specimen (Fig. 2) was captured in trammel nets (mesh size 36 mm, target species: cuttlefish), at a depth of 14 m on a muddy bottom and was examined onshore by fisheries observers. The catch also included two *D. pastinaca* individuals (Fig. 2a). The specimen, used in the present study, was donated by local fishermen, therefore a collection licence was not required. The marbled stingray specimen was brought to the fisheries laboratory of the HCMR, where it was identified according to Serena 2005, Bariche 2012, Séret 2016 and Last et al. 2016b.

Genomic DNA was extracted using the Chelex resin protocol (Walsh et al. 1991). DNA barcoding was applied by using the mitochondrial cytochrome c oxidase subunit 1 (COI) and the universal primer pairs (FistF2_t1 and FishR2_t1), following Ivanova et al. 2007 (Table 2). The polymerase chain reaction (PCR) cycling conditions for the ampliﬁcation included an initial denaturation at 94°C for 4 min, followed by 35 cycles at 94°C for 30 s, 52°C for 40 s, 72°C for 50 s and a ﬁnal extension at 72°C for 5 min. The PCR was conducted in 25 μl volumes and included 1.3 μl of DNA template, 5 μl GoTaq×5 reaction buffer (Promega), 1.5 μl of MgCl_2_ (1.5 mM), 200 μM of each deoxyribonucleotide triphosphate (dNTP; Promega), 0.5 μl (300 μM) of each primer, 1 U GoTaq G2 Flexi polymerase (Promega) and 14.5 μl molecular grade water. Subsequently, the PCR product was sequenced commercially (Macrogen, The Netherlands).

The sequence was compared with those available in GenBank using the standard nucleotide BLAST (blastn) against the nucleotide collection (nr/nt) database (http://blast.ncbi.nlm.nih.gov/Blast.cgi) and the BOLD database (Species Level Barcode Records, http://www.boldsystems.org).

## Taxon treatments

### Dasyatis marmorata

(Steindachner, 1892)

A39C719A-0311-5D12-B498-3FA2DAB02AE9

#### Materials

- **Type status:** Other material. **Occurrence:** occurrenceRemarks: collected dead in fishing nets; recordedBy: Stefanos Kavadas; individualCount: 1; sex: male; lifeStage: juvenile; reproductiveCondition: non-reproductive; preparations: whole animal, photographs, DNA extract; associatedSequences: GenBank: MT044303; **Taxon:** kingdom: Animalia; phylum: Chordata; class: Chondrichthyes; order: Myliobatiformes; family: Dasyatidae; genus: Dasyatis; taxonRank: species; vernacularName: marbled stingray; **Location:** continent: Europe; waterBody: Aegean Sea; country: Greece; countryCode: GR; municipality: Central Greece; locality: Maliakos Gulf; verbatimLatitude: 38.882326; verbatimLongitude: 22.592301; verbatimCoordinateSystem: decimal degrees; **Identification:** identificationID: 371208; identificationReferences: "Rays of the world. Last et al. 2016". "Chondrichthyans and Cyclostomata from the North-eastern Atlantic and the Mediterranean. Iglesias 2013".; identificationRemarks: blue blotches on pectoral fins, disc length vs. disc width; **Event:** samplingProtocol: trammel net; samplingEffort: 27 sampling hours; eventDate: 2019-02-09T09:00+0200; startDayOfYear: 39; endDayOfYear: 40; year: 2019; month: February; day: 9; habitat: muddy bottom; fieldNumber: 1902MLGT/T1H1/CM1; **Record Level:** type: By-catch entanglement; language: en; rightsHolder: Hellenic Centre for Marine Research; institutionCode: HCMR; basisOfRecord: Dead specimen

#### Description

The collected specimen was a juvenile male measuring 330 mm in total length, 160 mm in disc length and 188 mm in disc width and weighing 171.8 g (total weight). Additionally, twenty six morphological characters were also recorded (Table 1) following biometric measurements from recent studies (Psomadakis et al. 2008, Smith et al. 2009, Capapé et al. 2015) and the specimen was subsequently dissected. All collected field data and measurements were stored in the IMAS-fish database, a centralised integrated fisheries information system of the Institute of Marine Biological Resources and Inland Waters (Kavadas et al. 2013).

## Analysis

Morphological measurements were expressed as percentage of DW as previously proposed for sting rays (Cowley and Compagno 1993, Yeldan and Gundogdu 2018) and are presented in Table 1. The disc length to disc width ratio was 1.17, corroborating descriptions provided in Last et al. 2016b and Séret 2016. Additionally, the interorbital space to orbital length ratio was more similar to that reported for *D. marmorata* (1.5 times) than that from *D. pastinaca* (1.8-2 times) (Last et al. 2016b). Previous morphometric measurements from other areas involved larger specimens (mostly adult rays), thus our results should be interpreted with some caution as the analysis was based on a single specimen (one juvenile individual). A partial sequence of COI was generated (561 bp, GenBank Accession Number: MT044303). The barcode search on GenBank produced clear top matches with 100% similarity to *Dasyatis marmorata* records reported in the database. Additionally, BOLD comparisons showed similar matching rates. The molecular results obtained verified the macroscopic identification of the species.

## Discussion

The present study describes the first record of the marbled ray, *D. marmorata*, collected in Greek waters, extending its distribution further into the Eastern Mediterranean Sea. Morphological measurements and molecular tools were combined to identify the species in the studied area. This is consistent with recent records of *D. marmorata* along the Turkish coast (Bilecenoglu in Kapiris et al. 2014, Erguden et al. 2014, Özgür Özbek et al. 2015, Yeldan and Gundogdu 2018), while the absence of records from Greece could be attributed to the misidentification of the species and/or the limited surveys carried out in coastal areas (preferred habitats of *D. marmorata*). Species records from the latest book of fish fauna in Greece do not include the marbled ray (Papaconstantinou 2014) and other recent books do not extend its distribution in the Mediterranean Sea (e.g. "Rays of the World", Last et al. 2016b). Therefore, its distribution needs updating. Additionally, Maliakos Gulf (the origin of this specimen) appears to provide habitat for rare batoids in Greece (Kavadas and Siapatis in Ζenetos et al. 2015) and could be important for future studies on species richness, distribution and abundance of elasmobranchs. This kind of fundamental information on the distribution and habitat selection of rare and vulnerable chondrichthyan species is essential for their management and conservation (Saidi et al. 2016).

Most studies on the occurrence of elasmobranchs in the Mediterranean Sea have utilised and focused on the effects of trawl and longline fisheries (Follesa et al. 2019, Peristeraki et al. 2020), whilst the small-scale fisheries have often been overlooked, especially the action of by-catch (Saidi et al. 2016). This study emphasises the importance of scientific surveys using all types of fishing gear to assess the biodiversity in areas with huge knowledge gaps. This is especially true in the Eastern Mediterranean, where elasmobranch catch rates are lower compared to those reported in the Western Mediterranean (Damalas and Megalofonou 2012).

Biodiversity and distribution studies of the batoids are currently under investigation in Greece at the Institute of Marine Biological Resources of HCMR (Athens), in collaboration with the Fisheries Research Institute (Kavala). Priority should be given to rare and vulnerable species occurring in the Eastern Mediterranean, to improve their conservation and restrict further biodiversity loss. Furthermore, this study highlights the utility of DNA barcoding in assisting species identification and its role to accurately determine the occurrence and distribution of species.

## Supplementary Material

XML Treatment for Dasyatis marmorata

## Figures and Tables

**Figure 2a.**
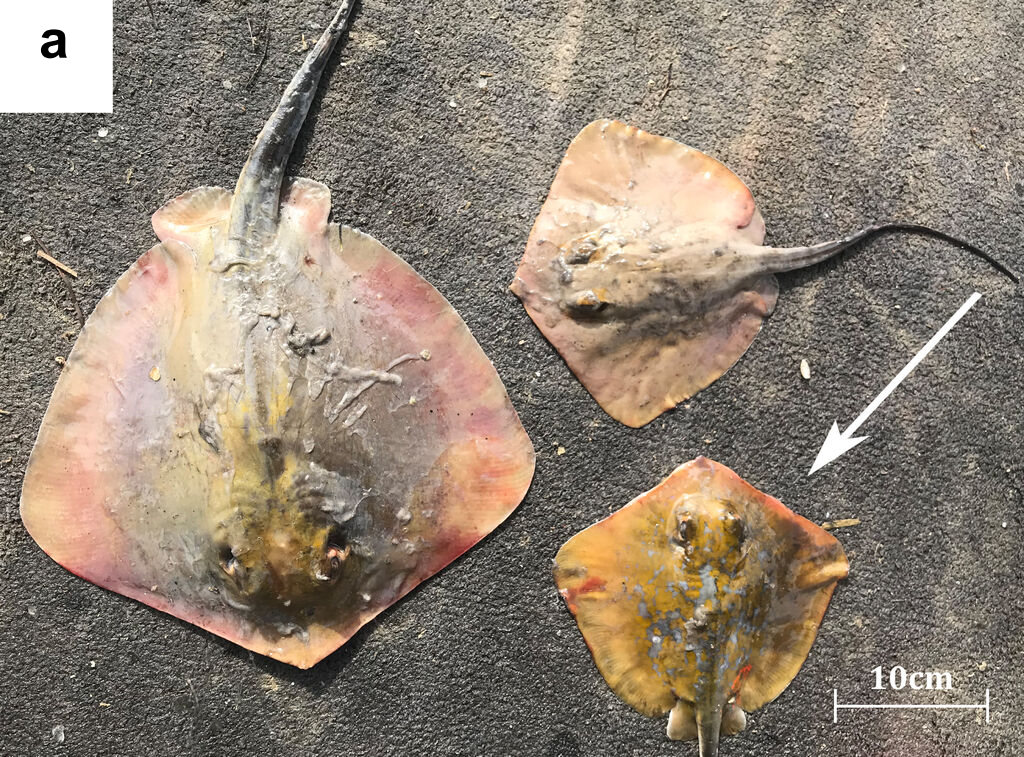
*Dasyatis marmorata* (with a white arrow) and two *D. pastinaca* specimens examined onshore.

**Figure 2b.**
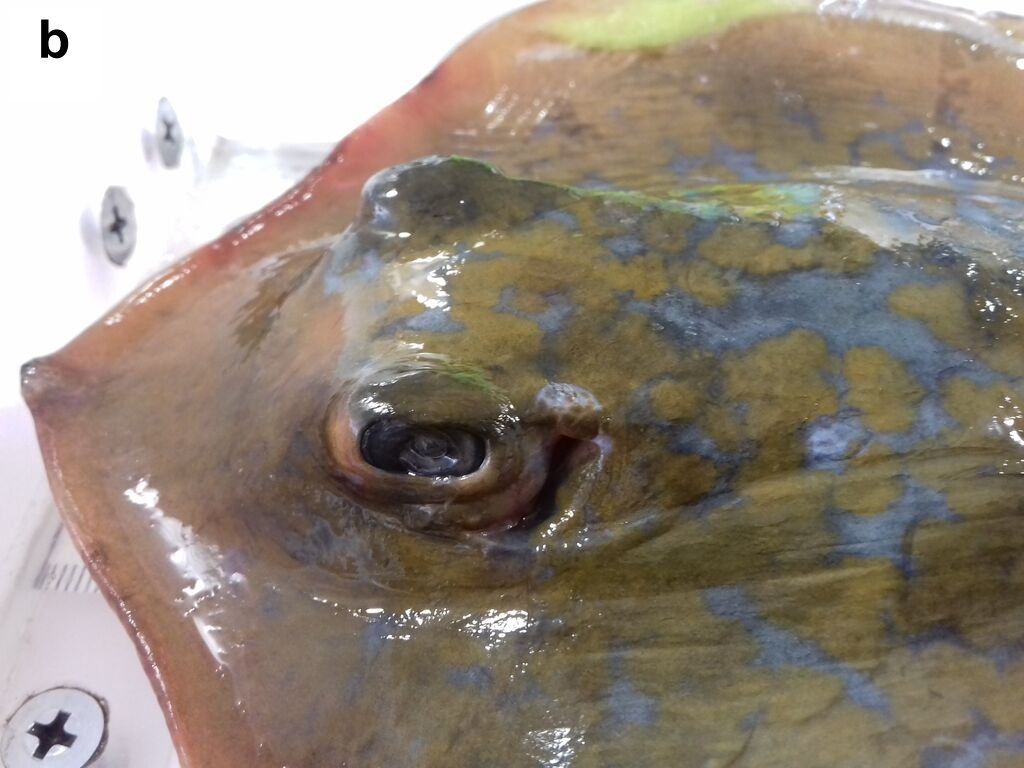
The distinctive blue blotches on the dorsal surface of the marbled ray.

**Figure 2c.**
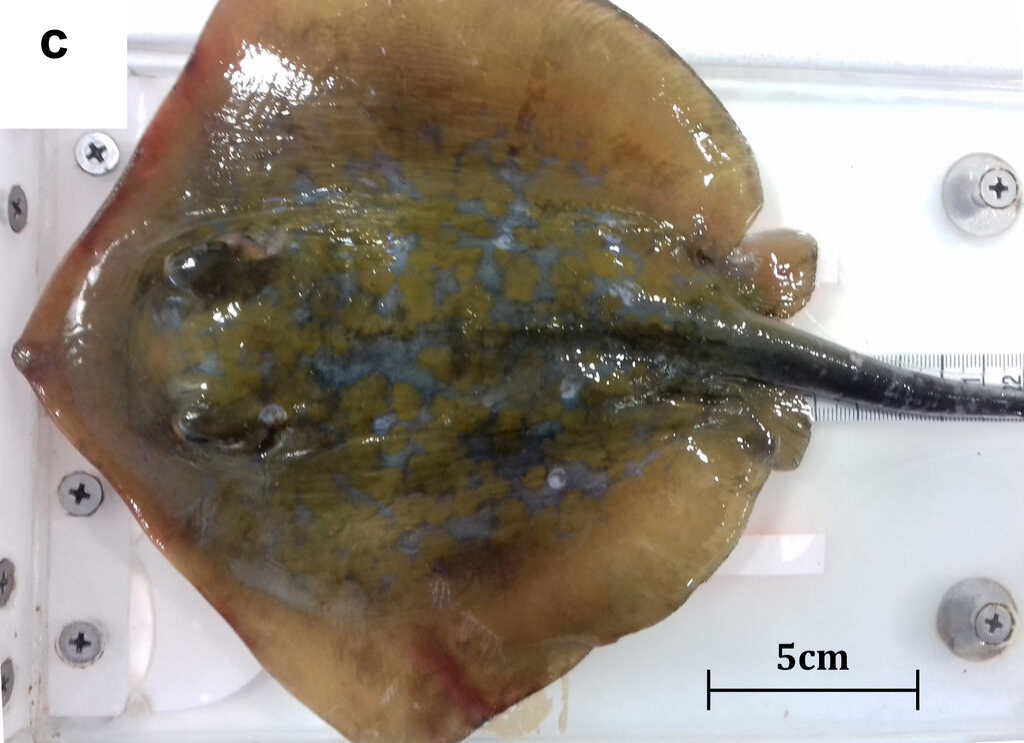
*Dasyatis marmorata*: dorsal view of the juvenile.

**Figure 2d.**
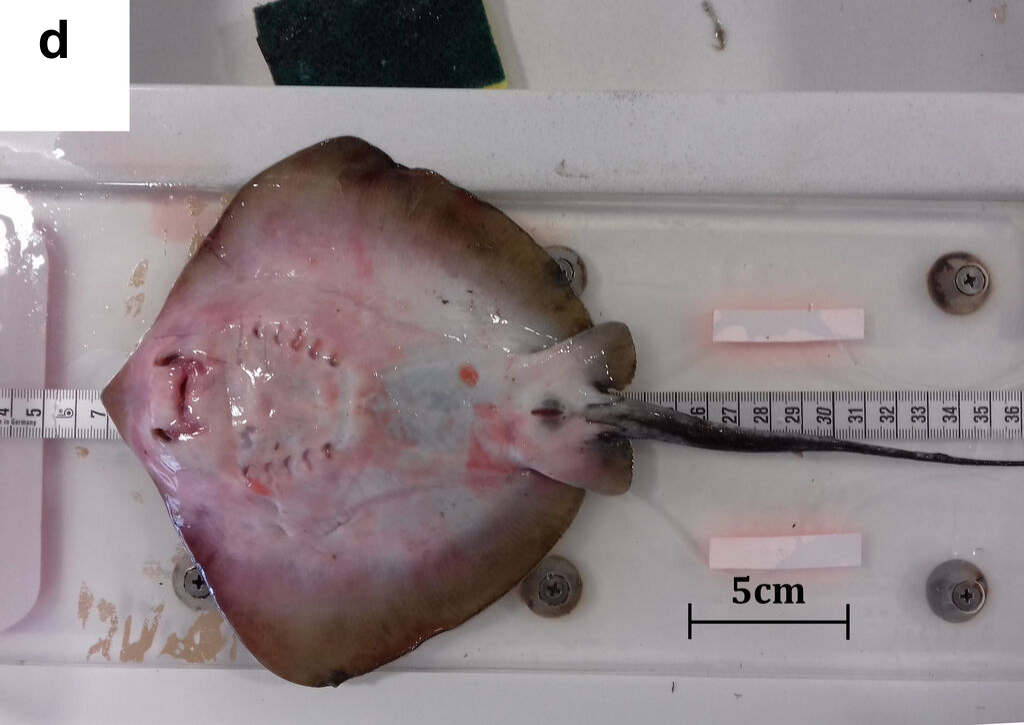
*Dasyatis marmorata*: ventral view of the juvenile.

**Table 1.** Morphological characters of *D. marmorata* from Maliakos Gulf, Greece and percentages against disc width (DW), following previously proposed biometric measurements. The total and eviscerated weight of the animal are also included in grams (g).

**Characters**	**mm**	% **to DW**	**g**
Total length	330.00	-	
Disc length	160.00	85.11	
Disc width	188.00	100.00	
Interorbital distance	27.11	14.42	
Interspiracular distance	32.15	17.10	
Orbit length	17.30	9.20	
Spiracle length	13.86	7.37	
Preorbital length	38.36	20.40	
Prespiracle length	52.17	27.75	
Pelvic anterior length	33.56	17.85	
Pelvic width	21.69	11.54	
Prenasal length	28.77	15.30	
Preoral length	37.37	19.88	
Internarial length	19.19	10.21	
Nasal curtain length	10.09	5.37	
Nasal curtain width	20.85	11.09	
Mouth width	20.86	11.10	
Distance between 1st gill slits	37.26	19.82	
Distance between 5th gill slits	24.00	12.77	
Snout to 1st gill length	54.61	29.05	
Width of tail at cloaca	13.25	7.05	
Width of tail at sting origin	6.76	3.60	
Snout to cloaca length	145.00	77.13	
Cloaca to tail tip length	195.00	103.72	
Cloaca to sting origin	79.61	42.35	
Pectoral anterior length	120.00	63.83	
Pectoral posterior length	115.00	61.17	
Clapser external length	7.71	4.10	
Clasper internal length	13.66	7.27	
Total weight			171.80
Eviscerated weight			151.60

**Table 2.** Sequencing primers used for species identification in this study.

**Primer**	**Primer sequence (5’-3’)**	**mtDNA target**	**Reference**
FistF2_t1	TGTAAAACGACGGCCAGTCGACTAATCATAAAGATATCGGCAC	COI	Ivanova et al. 2007
FistR2_t1	CAGGAAACAGCTATGACACTTCAGGGTGACCGAAGAATCAGAA		
